# Oral gabapentin for scalp pruritus in patients with lichen planopilaris: A case series

**DOI:** 10.1016/j.jdcr.2024.06.022

**Published:** 2024-07-05

**Authors:** Li-Chi Chen, Chino Ogbutor, Ümmügülsüm Yıldız-Altay, Kristen J. Kelley, Maryanne M. Senna

**Affiliations:** a Department of Dermatology, Lahey Hospital & Medical Center, Burlington, Massachusetts; b Department of Dermatology, University of Massachusetts Chan Medical School, Worcester, Massachusetts; c Department of Dermatology, Harvard Medical School, Boston, Massachusetts

**Keywords:** fibrosing alopecia in a pattern distribution, frontal fibrosing alopecia, gabapentin, itch, lichen planopilaris, pruritus

## Introduction

Lichen planopilaris (LPP) is a primary cicatricial alopecia (PCA) characterized by lymphocytic infundibulo-isthmic inflammation, resulting in the destruction of follicular bulge stem cells.^1^ Frontal fibrosing alopecia (FFA) and fibrosing alopecia in a pattern distribution (FAPD) are clinical variants of LPP.^2^ Scalp pruritus is a commonly reported symptom of LPP, potentially mediated by substance P (SP) and calcitonin gene-related peptide (CGRP) released from the dense sensory innervation of the inflamed bulge region.^3^ Gabapentin, an analog of gamma-aminobutyric acid that binds to the alpha_2_–delta subunit of the voltage-gated calcium channel, is suggested to have an antipruritic effect on PCA by suppressing neuronal hyperexcitability and subsequently reducing release of sensory neuropeptides.^4^ However, clinical evidence on the efficacy and safety of oral gabapentin for scalp pruritus in LPP patients has yet to be documented.

For this case series, we conducted a retrospective review on patients with LPP/FFA/FAPD treated with oral gabapentin for ≥3 months at a specialty alopecia clinic between January 2018 and January 2024. Gabapentin was added as a single agent or in combination with an additional topical therapy to the concurrent hair loss treatments. Concurrent treatments were initiated at least 3 months before the commencement of oral gabapentin, and the patient’s response to those concurrent treatments had reached a plateau. The patients were started on oral gabapentin with an initial dose of 100 mg daily at bedtime, with the option to increase the dose by 100 mg daily per week up to 300 mg daily based on patient symptomatology. If scalp pruritus persisted at the 3-6 months follow-up, the dose was further increased by the prescribing physician (MMS). Scalp pruritus and disease activity were evaluated using the itch numeric rating scale (NRS; range, 0-10 [no itch to worst imaginable itch]) and Lichen Planopilaris Activity Index (LPPAI; range, 0-10 [no disease activity to maximal disease activity]), respectively. Efficacy for scalp pruritus was classified as complete, partial, or no resolution of itch, defined as a reduction in itch NRS of 100%, 25% to 99%, and less than 25% compared to baseline, respectively. Additionally, the pretreatment and post-treatment itch NRS and LPPAI scores were compared using the Wilcoxon signed-rank test.

## Case series

**Fig 1:**
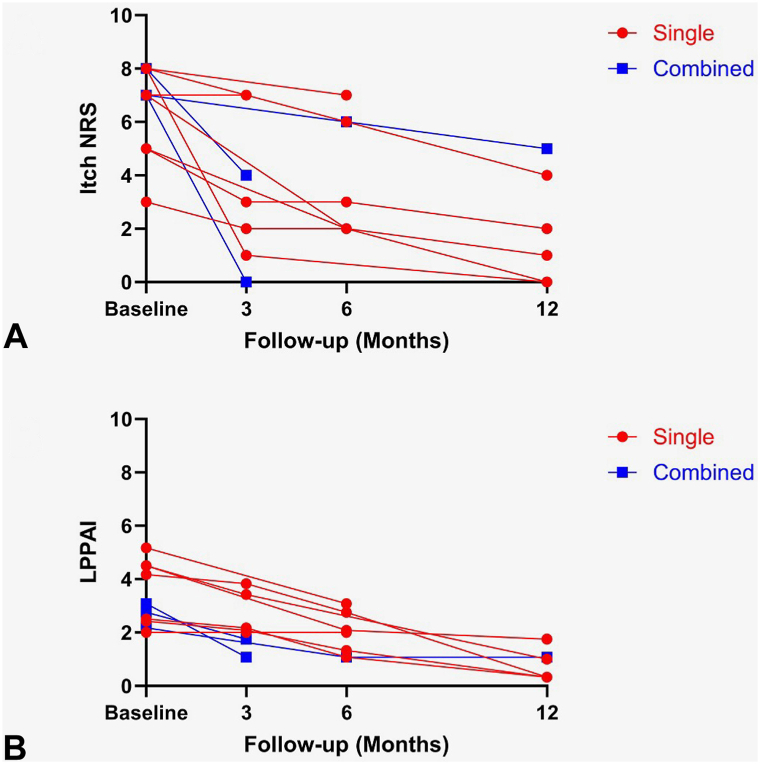
Spaghetti plots depicting trajectories of change in (**A**) itch numeric rating scale (NRS) and (**B**) Lichen Planopilaris Activity Index (LPPAI) over time with oral gabapentin, stratified by single or combined addition of oral gabapentin to patient’s concurrent hair loss therapies. Each line represents 1 subject.

Among 11 eligible patients with compliant use of gabapentin, all were Caucasian females with classic LPP (54.5%), FFA (36.4%), or FAPD (9.1%) (Table I). The mean age at diagnosis was 60.5 years (standard deviation [SD], 14.4), with a mean disease duration of 3.9 years (SD, 1.6). Gabapentin was incorporated into the existing therapies as a single (72.7%) or combined (27.3%) addition with a daily dose ranging from 100 to 900 mg. Overall, complete and partial resolution of scalp itch were achieved by 27.3% (3/11) and 54.5% (6/11) of patients, respectively. The patients exhibited a significant reduction in both itch NRS and LPPAI scores (mean pretreatment vs post-treatment itch NRS and LPPAI scores were 6.6 vs 2.9 and 3.21 vs 1.11, respectively; *P* = .006 and .009) with a mean 62% decrease in LPPAI score (Table II). Notably, this improvement was also observed in patients who initiated gabapentin as a single addition to their concurrent regimen (Table III, Fig 1). Among 3 patients with coexisting scalp burning, 2 (66.6%) reported diminution of burning sensation. Somnolence was reported in 1 (9.1%) patient, and 1 (9.1%) discontinued at 3 months for lack of efficacy. Oral gabapentin was otherwise well-tolerated.

**Table I:** Patient demographics, history at presentation, and previously attempted treatments

Case	Sex	Race	Age at diagnosis, y	Disease duration, y	LPP subtype	Diagnostic method	Previous treatments∗	Relevant comorbidities
1	F	White	71	6	FFA	Biopsy	Oral corticosteroid, TCS, TCI	−
2	F	White	69	6	Classic LPP	Biopsy	Topical gabapentin	CREST syndrome, mixed connective tissue disease, small fiber neuropathy
3	F	White	32	2	Classic LPP	Biopsy	Hydroxychloroquine, ILC	Pityriasis rosea
4	F	White	53	3	Classic LPP	Biopsy	Hydroxychloroquine, doxycycline, finasteride, pioglitazone	−
5	F	White	77	3	FFA	Biopsy	Doxycycline, TCS	Hypothyroidism
6	F	White	60	6	FFA	Clinical	Finasteride, TCS, ILC	−
7	F	White	72	3	FAPD	Biopsy	Minocycline, finasteride, TCS	Allergic rhinitis
8	F	White	35	2	Classic LPP	Biopsy	TCS, ILC	Prurigo nodularis, hypothyroidism
9	F	White	62	3	Classic LPP	Biopsy	Hydroxychloroquine, doxycycline, finasteride, naltrexone	−
10	F	White	73	6	FFA	Clinical	Topical tofacitinib, TCS, ILC	−
11	F	White	62	3	Classic LPP	Biopsy	TCI, ILC	−

*FAPD*, Fibrosing alopecia in a pattern distribution; *FFA*, frontal fibrosing alopecia; *ILC*, intralesional corticosteroid; *LPP*, lichen planopilaris; *TCI*, topical calcineurin inhibitor; *TCS*, topical corticosteroid.

∗ Previous treatments were discontinued prior to initiation of oral gabapentin.

**Table II:** Oral gabapentin treatment course, duration, and outcome in patients with lichen planopilaris

Case	Dose of gabapentin, mg	Single addition of gabapentin to the concurrent treatments∗	Duration of gabapentin use, months	Baseline itch NRS	Post-treatment itch NRS	Percentage change in itch NRS, %	Baseline LPPAI	Post-treatment LPPAI	Percentage change in LPPAI, %	Improvement of perifollicular inflammation	Concurrent treatments	Reported adverse events and discontinuation
1	300 QHS	Yes	28	8	0	−100	4.5	1	−77.8	Yes	Hydroxychloroquine, topical tofacitinib	−
2	300 QAM + 400 QHS	Yes	55	8	4	−50	2.5	0.33	−86.8	Stable†	Hydroxychloroquine, MMF, TCS, ILC	−
3	100 QHS	No‡	7	7	5	−28.6	2.17	1.08	−50.2	Stable†	TCS	−
4	100 QHS	No§	7	7	0	−100	2.75	0	−100	Yes	Oral minoxidil, TCI, ILC	Somnolence
5	100 QAM + 100 QHS	Yes	21	7	0	−100	4.5	1.75	−61.1	Yes	Finasteride, topical tofacitinib, spironolactone, ILC	−
6	100 QHS	Yes	67	8	7	−12.5	2	2	0	Stable†	Doxycycline, oral minoxidil, TCI	−
7	300 QHS	No‖	13	8	4	−50	3.08	0.33	−89.3	Yes	Topical tofacitinib, oral minoxidil, fexofenadine	−
8	100 QHS	Yes	3	7	7	0	2	2	0	Stable†	Topical minoxidil, TCI	Discontinuation at 3 mo for lack of efficacy
9	100 QHS	Yes	8	5	2	−60	5.17	3.08	−40.4	No	Oral minoxidil, TCS	−
10	200 QHS	Yes	60	5	2	−60	4.17	0.33	−92.1	Yes	Hydroxychloroquine, TCI	−
11	300 TID	Yes	36	3	1	−66.6	2.42	0.33	−86.4	Yes	Oral tofacitinib, TCI	−

*ILC*, Intralesional corticosteroid; *LPPAI*, Lichen Planopilaris Activity Index; *MMF*, mycophenolate mofetil; *NRS*, numeric rating scale; *TCI*, topical calcineurin inhibitor; *TCS*, topical corticosteroid.

∗ Single addition of oral gabapentin indicates that gabapentin was added as a single agent to existing treatments. Concurrent treatments in those patients were commenced at least 3 months before the initiation of oral gabapentin and the treatment response (measured by LPPAI and itch NRS) to those concurrent treatments had plateaued.

† Absence of perifollicular erythema or scale at baseline and after treatment with oral gabapentin.

‡ Oral gabapentin was added in conjunction with TCS to existing treatments.

§ Oral gabapentin was added in conjunction with TCI to existing treatments.

‖ Oral gabapentin was added in conjunction with topical tofacitinib to existing treatments.

**Table III:** Outcome of patients with lichen planopilaris treated with oral gabapentin for a minimum of 3 months

Itch Numeric Rating Scale (NRS)							
	*N*	Mean pretreatment itch NRS score (range)	Mean post-treatment itch NRS score (range)	*P* value	Complete resolution of itch∗, *n* (%)	Partial resolution of itch∗, *n* (%)	No resolution of itch∗, *n* (%)
All	11	6.6 (3-8)	2.9 (0-7)	.006	3 (27.3)	6 (54.5)	2 (18.2)
Single addition of gabapentin to concurrent treatments†	8	6.4 (3-8)	2.9 (0-7)	.022	2 (25.0)	4 (50.0)	2 (25.0)
Combined addition of gabapentin to concurrent treatments	3	7.3 (7-8)	3.0 (0-5)	—‡	1 (33.3)	2 (66.7)	0 (0)

Lichen Planopilaris Activity Index (LPPAI)					
	*N*	Mean pre-treatment LPPAI (range)	Mean post-treatment LPPAI (range)	*P*-value	Mean percentage change in LPPAI (range)
All	11	3.21 (2-5.17)	1.11 (0-3.08)	.009	−62.2 (−100 to 0)
Single addition of gabapentin to concurrent treatments†	8	3.41 (2-5.17)	1.35 (.33-3.08)	.036	−55.6 (−92.1 to 0)
Combined addition of gabapentin to concurrent treatments	3	2.67 (2.17-3.08)	0.47 (0-1.08)	—‡	−79.8 (−100 to −50.2)

∗ Complete resolution of itch: 100% itch NRS reduction compared to baseline; partial resolution of itch: 25% to 99% itch NRS reduction compared to baseline; no resolution of itch: <25% itch NRS reduction compared to baseline.

† Single addition of oral gabapentin indicates that gabapentin was added as a single agent to existing treatments. Concurrent treatments in those patients were commenced at least 3 months before the initiation of oral gabapentin and the treatment response (measured by LPPAI and itch NRS) to those concurrent treatments had plateaued.

‡ Insufficient sample size to conduct statistical analysis.

## Discussion

The pathogenesis of LPP has been linked to neurogenic inflammation given the decreased epidermal nerve fiber density and altered expression of SP and CGRP in the LPP-affected scalp, which may lead to small-fiber neuropathy and associated scalp symptoms.^5^ Gabapentin secondarily reduces the release of SP and CGRP from primary afferent neurons by primarily increasing gamma-aminobutyric acid release.^4^ Additionally, by binding to the voltage-gated calcium channel, gabapentin inhibits presynaptic calcium influx and glutamate release, thereby suppressing pruritic stimulation at trigeminal and cervical nerves.^6^^,^^7^ The antineuroinflammatory properties of gabapentin may diminish scalp pruritus and perifollicular inflammation alongside concurrent therapies, leading to a reduction in LPPAI. Topical or oral gabapentin (10% cream 3 times daily or 200-300 mg daily) was reported to alleviate scalp pruritus in patients with scalp dysesthesia in a small-scale retrospective study.^8^ A clinical trial is underway to examine the efficacy of topical 6% gabapentin (NCT03346668) for symptomatic PCA. Interestingly, oral gabapentin 300 mg 3 times daily has also demonstrated efficacy in treating hot flashes, a condition frequently seen in patients with LPP—which predominantly affects postmenopausal women.^9^ Furthermore, gabapentin has a broad therapeutic index, desirable safety profiles, high bioavailability, and minimal drug-drug interactions,^10^^,^^11^ which may be favorable for LPP patients who are often older and on multiple medications due to the challenging management.^12^^,^^13^

Our findings indicate the therapeutic potential of oral gabapentin for scalp pruritus in LPP, which is an essential component of disease activity measured by LPPAI. Limitations of this study include retrospective and noncontrolled design and adjunctive use of gabapentin in most patients. Larger prospective controlled trials are needed to confirm these findings.

## Conflicts of interest

None disclosed.
